# Development and formative evaluation of the e-Health Implementation Toolkit (e-HIT)

**DOI:** 10.1186/1472-6947-10-61

**Published:** 2010-10-18

**Authors:** Elizabeth Murray, Carl May, Frances Mair

**Affiliations:** 1 E-Health Unit, Research Department of Primary Care and Population Health, University College London, Upper Floor 3, Royal Free Hospital, Rowland Hill Street, London NW3 2PF, UK; 2 Faculty of Health Sciences, University of Southampton, University Road, Southampton, SO17 1BJ, UK; 3 Section of General Practice & Primary Care, Centre for Population and Health Sciences, University of Glasgow, 1 Horselethill Road, Glasgow G12 9LX, UK

## Abstract

**Background:**

The use of Information and Communication Technology (ICT) or e-Health is seen as essential for a modern, cost-effective health service. However, there are well documented problems with implementation of e-Health initiatives, despite the existence of a great deal of research into how best to implement e-Health (an example of the gap between research and practice). This paper reports on the development and formative evaluation of an e-Health Implementation Toolkit (e-HIT) which aims to summarise and synthesise new and existing research on implementation of e-Health initiatives, and present it to senior managers in a user-friendly format.

**Results:**

The content of the e-HIT was derived by combining data from a systematic review of reviews of barriers and facilitators to implementation of e-Health initiatives with qualitative data derived from interviews of "implementers", that is people who had been charged with implementing an e-Health initiative. These data were summarised, synthesised and combined with the constructs from the Normalisation Process Model. The software for the toolkit was developed by a commercial company (RocketScience). Formative evaluation was undertaken by obtaining user feedback.

There are three components to the toolkit - a section on background and instructions for use aimed at novice users; the toolkit itself; and the report generated by completing the toolkit. It is available to download from http://www.ucl.ac.uk/pcph/research/ehealth/documents/e-HIT.xls

**Conclusions:**

The e-HIT shows potential as a tool for enhancing future e-Health implementations. Further work is needed to make it fully web-enabled, and to determine its predictive potential for future implementations.

## Background

E-health, or the use of information and communication technology in health care, is seen as essential for a modern, cost-effective health service which is capable of addressing challenges such as improving equity of access and quality of care in a world facing an increasing burden of chronic disease [1]. There is an international commitment to e-Health, reflected in very considerable expenditure. The UK government has invested £12.4 bn over 10 years [2] and this is less than the US or many European countries [3]. However, despite the overwhelming political commitment and substantial investment, there has been significant variability in the success of different e-health implementations [4,5]. Many projects have been subject to delay [6], increasing budget overspends, and in some cases, severely negative impacts on the quality and effectiveness of care [7-9]. Although there is a considerable body of research on implementation of e-health [10,11], recent work has criticised both the methodology used in many of the existing reviews of this work, and the narrow focus on organisational issues related to implementation, with little attention paid to the impact of new technologies on workload, inter-professional relationships, and communication between health professionals and patients [12].

There are many reasons for the difficulties encountered with implementation of e-health. Some of these are likely to parallel those contributing to the gap between research findings and routine clinical care, including a perceived lack of relevance of research to practitioner needs [13]; managers, or other senior staff charged with e-Health implementations not having the time or inclination to read the large body of literature [14]; inadequacies in the existing research [12]; and the poor permeability of the managerial: research interface[15].

In this paper we describe the development and formative evaluation of an e-Health Implementation Toolkit (e-HIT) (Additional file 1). The e-HIT was developed explicitly to bridge the managerial:research interface, by making the results of a large body of research into e-Health implementation accessible to senior managers in a simple, user-friendly format. Its development had been explicitly commissioned by the funding body (the National Institute of Health Research Service Delivery and Organisation programme) as part of an overall programme of work examining the barriers and facilitators to e-health implementation in the UK National Health Service. The aim of the e-HIT was to summarise and synthesise research evidence on factors that impede or facilitate implementation of e-Health initiatives and present this evidence in a format that could be easily digested and used by staff considering or planning an e-Health implementation. The aim of this paper is to describe the process of development and formative evaluation, and describe the final toolkit, in line with recent calls for more detailed descriptions of the processes and content of complex interventions [16,17].

## Implementation

**Figure 1:**
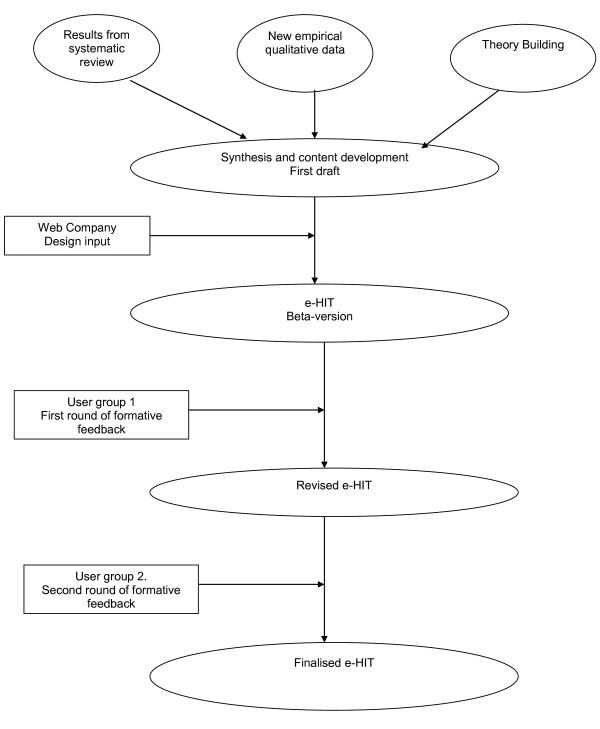
**Flow chart of e-Hit development**.

There were three phases to the development and formative evaluation of the e-HIT (Figure 1):

1. Writing the content;

2. Developing the excel spreadsheet;

3. Obtaining and incorporating user feedback on the toolkit.

### Writing the content

The content of the e-HIT was derived by combining a theoretical framework with a literature review and new empirical data.

#### The Normalisation Process Model

The theoretical framework was the Normalisation Process Model (NPM) which is a sociological model explaining why some new technologies become fully embedded in routine practice (normalised) and others do not. It was initially derived from an extensive series of qualitative studies of telemedicine initiatives [18], and subsequently refined and expanded to apply to the implementation of all complex interventions in health care. The NPM suggests that the degree of "normalisation" of a complex intervention, such as a new e-health technology, depends on the impact of the proposed intervention on four constructs[19]. These constructs are known as interactional workability, relational integration, skill set workability and contextual integration. Interactional workability (IW) refers to the degree to which the proposed technology enables (or impedes) interactions between health professionals and patients - e.g. a consultation. Relational integration (RI) refers to the way in which different professional groups relate to each other, and how well the proposed technology fits (integrates) with existing relationships, as well as the degree to which it promotes trust, accountability and responsibility in inter-group relationships. Skill set workability (SSW) refers to the degree to which the e-health initiative fits with existing working practices, skill sets, and perceived job role. Contextual integration (CI) refers to the degree to which the proposed e-health system fits (integrates) with the overall goals and structure of the organization (context), as well as the capacity of the organization to undertake the implementation. The more positive the impact of the proposed new technology on these four constructs, the more likely it is that it will normalise [19].

#### The literature review

A systematic "review of reviews" was undertaken, with the aim of summarising the available literature on barriers and facilitators to implementation of e-health systems. The methods and findings of this review have been described in detail elsewhere [12]. We searched MEDLINE, EMBASE, CINAHL, PSYCINFO and the Cochrane library for reviews of e-Health implementation. Inclusion criteria for this review were that the paper a) was a review; b) was on e-Health; and c) included information pertinent to implementation. For the purposes of this study we defined a review as providing an analytic account of the research literature, and included systematic reviews, narrative reviews and qualitative meta-syntheses. E-health was defined as "the use of emerging information and communications technology, especially the Internet, to improve or enable health and health care"[20], while implementation was defined as "all activities that occur between making an adoption commitment and the time that the innovation either becomes part of the organisational routine, ceases to be new, or is abandoned" [21]. The search strategy combined the two concepts of e-health and implementation and was limited by publication type to reviews. Titles and abstracts generated by the search strategy were downloaded into an electronic data-base and screened by two independent reviewers. Papers that could not definitely be excluded at this stage were obtained in full, and read by two independent reviewers who determined their eligibility against pre-determined inclusion and exclusion criteria. Data were extracted by two independent reviewers and subjected to a thematic analysis.

The searches yielded 6,585 citations of which 6,439 could be excluded on the basis of the title or abstract. 146 full papers were retrieved, of which 19 met the criteria for inclusion. The thematic analysis found that the existing literature focused on the following factors: conditions prior to implementation; costs of implementation; importance of evaluation of the new technology prior to implementation; attitudes of users, particularly health professionals; ease of use of the system; security, confidentiality and standards; education and training; technological issues; communication; and organisational issues. Conditions prior to the implementation had two components - those within the organisation under study, and the broader societal context. Examples pertaining to the specific organisation included pre-existing working relationships, morale, and previous experience of e-health implementations, while the broader societal context could include policy or economic drivers. Costs, including the costs of obtaining the system and the costs of implementation were seen as important, while some authors mentioned the costs of not implementing the system as also being relevant. Many authors commented on the importance of evaluation of systems prior to their implementation. Professional attitudes were often perceived as a barrier to implementation, which might or might not be overcome through education and training. System specific factors included ease of use, and whether concerns about confidentiality, security and standards had been adequately met. Technological issues were important, in terms of fitness for purpose, as was maintaining good communication both within an organisation and between professionals and patients [12].

#### Empirical data

The third source for the content of the e-HIT was newly obtained qualitative data, derived from interviews with a range of "implementers" with responsibility for one or more e-health implementations. The methods and results of this study have also been described in detail elsewhere [12]. "Implementers" were defined as any person charged with assisting with the implementation of an e-health system. We included chief executives of trusts, clinical directors, senior managers, ICT staff, and staff working for private companies contracted to supply, deliver or facilitate specific e-health implementations. We selected three case studies, which between them covered a range of NHS contexts (primary, community and secondary care), types of e-health technology and relationship with the main sponsor in this arena, namely Connecting for Health. The selected studies were Choose and Book in one hospital trust and its main referring Primary Care Trust in inner London, Picture Archiving and Communication System in a hospital trust with several sites in South West England, and a Clinical Nurse Information System in a large urban health board in Scotland. In all cases the implementation had happened between 12 and 36 months earlier. Interviewees were asked for their perceptions of barriers and facilitators to the implementation of the study technology, and the degree to which the technology had normalised. Data were coded by two independent researchers, using the NPM as a coding framework. The results suggested that the NPM provided a good explanation for the variable degrees of normalisation of the three study technologies. E-health initiatives that were perceived by respondents to have a positive impact on all four constructs were highly likely to normalise. Problems with any one construct should alert policy makers and senior managers to potential difficulties, which need careful consideration and planning. Difficulties across all four constructs suggest that the initiative is relatively unlikely to normalise, and some rethink may be needed [12].

#### Combining the data sources

In order to create the content of the e-HIT, the main themes that arose from the literature review and qualitative study were considered in depth, along with the constructs of the NPM. The themes were synthesised to create a database of items which had theoretical and empirical validity. In order to make this collection of items accessible and comprehensible, they were grouped into three main categories: the context; the intervention; and the workforce. These categories emerged from the interview data from implementers, and were selected as resonating with the approach currently in use by implementers. The next task was to reduce the number of items in each group to the minimum compatible with adequately addressing all the issues which were judged important on the basis of the three data sources (literature review, qualitative data and theoretical framework). This was done by reviewing all the items, deleting duplicates, and clarifying items which were ambiguous. Subsequently, each item was operationalised by a statement, which was anchored by extreme negative and extreme positive positions.

### Developing the toolkit

We contracted a web design company (RocketScience) who had experience of building toolkits for the public and private sectors (including the NHS) to provide the software for the toolkit. We undertook an iterative process of discussion and design which culminated in a prototype toolkit, ready for user testing and feedback. Design issues considered in this phase included the number of points on the scale between anchor points for each statement, the number of items on each page, the number of pages, the use of colour, page layout, navigation, and the presentation of results once the toolkit had been completed. The web design company's previous experience was invaluable at this stage.

Additional material was written to inform users how the toolkit had been derived, its aim, how to download and complete it, and how to interpret the results. The section on interpreting the results emphasised that these should be used to alert senior managers to potential problems and encourage constructive, organised thinking about the implementation process, not a tickbox or scorecard approach which could replace careful thought.

Finally, we provided three illustrative case studies for users to look at, and compare with their own planned implementation. These case studies were those used in the qualitative study described above.

### User feedback

A two stage formative evaluation of the e-HIT was undertaken. For the first stage, the prototype e-HIT was circulated to a group of e-Health experts. These experts included senior clinicians, managers and academics each of whom had extensive experience of e-health implementations within the NHS (n = 13). These experts were asked to complete the e-HIT for an e-Health initiative they had personal experience of, and on the basis of this experience, to comment critically on the e-HIT. Specifically, respondents were asked whether they thought the e-HIT would be useful to senior managers considering, planning or undertaking an e-Health implementation, what would make the e-HIT more useful, what were the positive features of the e-HIT, and what features needed modifying. Respondents were asked to suggest modifications which would improve the e-HIT, and for other general or specific comments not covered by the questions listed (Additional File 2).

All the experts contacted responded with comments and discussion. The overall response was overwhelmingly positive, with comments including "fantastic" and "excellent piece of work". Specifically, respondents thought that the non-prescriptive approach and emphasis on the e-HIT as a sensitizing tool would appeal to senior managers. Respondents thought that the overall layout was clear, the language easily comprehensible, and that the main areas of importance were well covered. They liked the sliding scales, space for explanatory text, and instant feedback. There were also specific suggestions for improvement about the navigation, layout and wording of individual components of the e-HIT, with requests for more information to help with completing some of the questions, and clarification of individual questions. The experts raised two major concerns which we were unable to address within the constraints of the resources available within this research project: a) they judged that the e-HIT would be more useful if it was fully web-enabled, which would allow multiple users to compare their results, and hence share experience either between different professional groups or across multiple implementation sites; and b) there was a concern about how best to disseminate the toolkit.

This feedback led to a number of changes, including redesign of the introductory section, allowing experienced users to bypass this, provision of explanatory mouseovers to assist with completion of individual questions, a more streamlined lay-out and a more detailed explanation of how to use the report section of the toolkit.

In the second stage of the formative evaluation, the revised toolkit was circulated by e-mail to the implementers who had been interviewed in the qualitative study (n = 23). The e-mail explained that the toolkit had been generated in part from their interview data, and that we were aiming to make the toolkit as helpful as possible to people like them. Participants were asked to comment on the likely usefulness of the e-HIT, make suggestions for improvement, and whether it adequately reflected their own experience (Additional file 3). This round of feedback elicited only minor suggestions from 5 respondents, e.g. further clarification of specific items.

## Results

There are three components to the toolkit - a section on background and instructions for use aimed at novice users; the toolkit itself; and the report generated by completing the toolkit. The background and instructions section can be bypassed, but for those who wish to use it, it contains advice on how to use the toolkit, including what it is, who should use it, when it should be used, and how it should be used. This section emphasises that it is a sensitising tool to alert the user to potential pitfalls and allow pre-emptive planning. There is a short section on how the toolkit was derived, including links to further reading for those interested. This introductory section also contains links to the reports generated by the illustrative case studies, to give users an idea of what the report will look like, and how it compares with their own experience.

The toolkit itself has 6 pages: 3 pages on context, 1 on the intervention and 2 on workforce issues. Context includes organisational factors, national and local policies, and other drivers of the implementation. Intervention items address the impact on professional - patient interactions, inter-professional relationships, and the effectiveness and cost-effectiveness of the intervention. Workforce items consider the impact of the intervention on workload, workflow, distribution of work between different user groups, the need for education and training, and the impact on relationships between professional groups. This grouping of items was intended solely to help organise and present the items as it was recognised that some items could be considered to belong in two or more categories.

**Figure 2:**
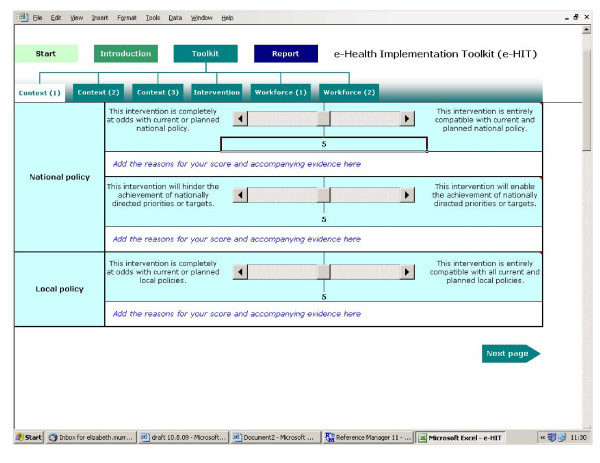
**Screen shot of sample page**.

Each page contains three items with an extreme negative and an extreme positive anchoring statement. The user is asked to rate their proposed e-health implementation on a scale of 0 - 10 (Figure 2).

The final section of the toolkit consists of the report it generates once the questions have been answered. There are four parts to the report: guidance on how to use the report; graphical displays of the responses for each item; a summary of the comments made by the user; and a problems and solutions page where the user can note potential areas of difficulty in the proposed implementation and plans for addressing these. To see one of the illustrative case reports, please see: http://www.ucl.ac.uk/pcph/research/ehealth/documents/case_study-PACS.xls#Scores!A4

## Discussion

To the best of our knowledge, this is the first e-Health Implementation Toolkit to be developed in this rigorous way. The e-HIT has a number of strengths, including its theoretical underpinning, in line with the call to improve the design of implementation interventions with the use of theory [22]. Undertaking a systematic review of reviews of the field of e-health implementation allowed us to synthesise and summarise a very large body of literature in a reasonably efficient manner. In addition we obtained new qualitative data, from a group who have been relatively understudied, namely implementers, or people charged with an e-health implementation. Including these data helped ensure that the toolkit incorporated the perceptions of the professional group the toolkit is aimed at. The toolkit was subjected to constructive criticism from a team of experts in the field. These strengths in its development may account for the very positive response obtained during the formative evaluation. However, there are a number of weaknesses, and it would be premature to advise widespread use of the e-HIT until research into its effectiveness has been undertaken. Weaknesses include the relatively small number of people who have used and commented on the e-HIT, and, as highlighted during our formative evaluation, it would be more useful if fully web-enabled. It is also unclear how useful the e-HIT will be outside the UK National Health Service. The literature review reported experience gained internationally, but the additional qualitative data obtained from implementers, and the case studies are UK-based.

We believe that the e-HIT has the potential to meet an unmet need in the NHS as previous toolkits tend to have either focused on readiness to implement research findings (e.g. those derived from the Promoting Action on Research Implementation in Health Services (PARiHS) framework [23,24])) or on supporting quality improvement interventions in health care [25]. Lilley and Navein developed "*The Telemedicine Toolkit" *in 2000 [26], but this focuses exclusively on telemedicine and is a workbook of 185 pages with exercises. We are seeking further funding to web-enable the toolkit, and subject it to further critical evaluation, including determining its applicability in an international context.

## Conclusions

The e-HIT is a new tool which has potential for enhancing and improving e-Health implementations. More work is needed to web-enable the toolkit, and to determine its potential predictive power.

## Availability and requirements

The e-HIT is available for downloading from http://www.ucl.ac.uk/pcph/research/ehealth/documents/e-HIT.xls. There are no restrictions on use.

## Abbreviations

e-HIT: e-Health Implementation Toolkit

## Competing interests

The authors declare that they have no competing interests.

## Authors' contributions

EM led on developing the e-HIT with support from CM and FM. EM wrote the first and final drafts of the manuscript with input from CM and FM. All authors have made substantial contributions to the concept and design of the study and to the drafting of the manuscript. All authors have read and agreed the final draft.

## Pre-publication history

The pre-publication history for this paper can be accessed here:

http://www.biomedcentral.com/1472-6947/10/61/prepub

## Supplementary Material

Additional file 1 **The e-HIT**. http://www.ucl.ac.uk/pcph/research/ehealth/documents/e-HIT.xls. Downloadable excel spread sheet of e-HIT. Click here for file

Additional file 2 **feedback questionnaire for Round 1**. Click here for file

Additional file 3 **feedback questionnaire for Round 2**. Click here for file
