# Supplementary material for: Development and formative evaluation of the e-Health Implementation Toolkit (e-HIT)
Source: BMC Med Inform Decis Mak. 2010 Oct 18;10:61. doi: 10.1186/1472-6947-10-61 (PMC2967499; doi:10.1186/1472-6947-10-61)
Supplement: Additional file 2 — feedback questionnaire for Round 1. [file 1472-6947-10-61-S2.DOC]

**Feedback form for first round of comments on the e-HIT.**

Name:

1. Do you think this type of approach is likely to be useful to the target audience (senior managers in the NHS planning an e-health implementation)? If so, why, and if not, why not? Do you have an alternative suggestion?

2. What features of the e-HIT do you think work well, and why? Please differentiate between presentation / navigation and content.

3. What features of the e-HIT do you think need changing? Why? And what suggestions for improvement do you have? Please differentiate between presentation / navigation and content.

4. Are there any specific, minor or editorial type comments you wish to make? Please differentiate between presentation / navigation and content.

Thank you very much for your help!

Elizabeth Murray
