# Supplementary material for: Development and formative evaluation of the e-Health Implementation Toolkit (e-HIT)
Source: BMC Med Inform Decis Mak. 2010 Oct 18;10:61. doi: 10.1186/1472-6947-10-61 (PMC2967499; doi:10.1186/1472-6947-10-61)
Supplement: Additional file 3 — feedback questionnaire for Round 2. [file 1472-6947-10-61-S3.DOC]

**Appendix 2: Feedback form for second round of comments on the e-HIT**

Name (optional):

1. Overall, do you think this toolkit is likely to be useful to the target audience? (People like you who will be involved in planning and implementing an e-health initiative within the NHS in the future). Why?

2. What features of the e-HIT do you think work well, and why?

3. What features of the e-HIT do you think need changing? Why? And what suggestions for improvement do you have?

4. Do you think this toolkit adequately captures most of the issues that you raised during your interview? Is there anything important that has been left out? Or anything included that should not be?

Thank you very much for your help!

Elizabeth Murray
